# Beyond Zipf’s Law: The Lavalette Rank Function and Its Properties

**DOI:** 10.1371/journal.pone.0163241

**Published:** 2016-09-22

**Authors:** Oscar Fontanelli, Pedro Miramontes, Yaning Yang, Germinal Cocho, Wentian Li

**Affiliations:** 1 Departamento de Matemáticas, Facultad de Ciencias, Universidad Nacional Autónoma de México, México, DF, México; 2 Bioinformatics Group and Interdisciplinary Center for Bioinformatics, University of Leipzig, Leipzig, Germany; 3 Department of Statistics and Finance, University of Science and Technology of China, Hefei, Anhui, China; 4 Instituto de Física, Universidad Nacional Autónoma de México, México, DF, México; 5 The Robert S. Boas Center for Genomics and Human Genetics, The Feinstein Institute for Medical Research, Northwell Health, Manhasset, NY, United States of America; Shanxi University, CHINA

## Abstract

Although Zipf’s law is widespread in natural and social data, one often encounters situations where one or both ends of the ranked data deviate from the power-law function. Previously we proposed the Beta rank function to improve the fitting of data which does not follow a perfect Zipf’s law. Here we show that when the two parameters in the Beta rank function have the same value, the Lavalette rank function, the probability density function can be derived analytically. We also show both computationally and analytically that Lavalette distribution is approximately equal, though not identical, to the lognormal distribution. We illustrate the utility of Lavalette rank function in several datasets. We also address three analysis issues on the statistical testing of Lavalette fitting function, comparison between Zipf’s law and lognormal distribution through Lavalette function, and comparison between lognormal distribution and Lavalette distribution.

## Introduction

It is said that a certain quantity follows a power law if the probability of observing it varies inversely as a power of this quantity. Power laws in data collected from natural or social phenomena are well documented [1]. For instance, the asymptotic occurrence of power laws in critical phenomena and statistical physics has been widely studied [2]. In the same way, power law tails have been reported in the distribution of word frequency [3], city sizes [4], fluctuations in financial market indexes [5], firm sizes in the U.S [6], scientific citations [7, 8] et caetera. Power laws are also observed in epidemic systems: beginning with the observation that epidemic sizes and durations are well characterized by power laws [9], this scale free behavior has been used to model patch sizes during an epidemic spread [10], as well as other relevant spatial patterns in theoretical ecology [11]. There are two common approaches in displaying a power law distribution: the histogram, which approximates the probability density function (pdf), and the rank-frequency plot, best known by the Zipf’s law for usage of words in human languages [3, 12].

Empirical data often exhibit good power-law distribution within a limited range, whereas one or both ends of the distribution may deviate from the ideal power law [13]. It is a well known fact that any finite size system, that is well described by a power law, deviates from this behavior due to finite size effects [14]. In these systems, the power law ceases to hold in a certain region, where effects due to the finiteness of the system dominate the behavior (for example, finite sample size or finite available energy). Therefore, it is natural to see deviations from power laws at the tails. However, the question remains of whether deviations are merely explained by finite size effects or if they call for a modification in the whole body of the distribution. This paper explores the second possibility. Modifying a power law by changing the functional form potentially may fit the systematic deviation. Previously, we proposed a rank-frequency function, inspired by the Beta density function [15], called Beta-like function [16], or Discrete Generalized Beta Distribution (DGBD) [17], or Cocho rank function [18]. The DGBD

$$x_{\left[r\right]}=C\frac{{\left(N+1-r\right)}^{b}}{r^{a}}$$ (1)

(*x*: quantity of interest, *r*: rank, *N*: the maximum rank), contains the fitting parameters *a* and *b* and the normalization factor *C*. We previously proposed that the parameter *a* is associated with the behavior which leads to the power law, whereas *b* is associated with the fluctuation in noise [17]. An example of the former is the inertial range in turbulence where energy is transferred between different length scales with the same rate, while an example of the latter is the dissipative range in turbulence [17]. Another example is in a conflicting dynamics called expansion-modification systems [19], where *a* > *b* when expansion dominates mutation and *b* > *a* when mutation dominates [20]. Eq (1) modifies the power law rank function 1/*r*^*a*^ by a power of the reverse-rank *r*_2_ = *N* + 1−*r*, and it converges to power law when *b* = 0. DGBD often surpasses other two-parameter functions in fitting real data [7, 18, 21], and achieved various degree of success in other applications [7, 16, 17, 22–27].

It is a well known fact that a quantity that follows a power law in the rank-frequency representation has a Pareto distribution [28]. The widespread application of the DGBD raises the issue of whether it is the result from a well known pdf, such as the normal/Gaussian distribution. In this work, we show that for a special case of the DGBD, the Lavalette rank function where *a* = *b* [29–33], the corresponding pdf can be derived analytically. The Lavalette rank function is also intrinsically connected, by an approximation, to the lognormal distribution. We offer both numerical evidence and an analytic proof.

The paper is organized in the following way: first we derive and characterize the pdf associated with the Lavalette rank function, which we call the Lavalette distribution, and show that it is approximately equal to the lognormal distribution over a relatively large interval. Next we exhibit applications of the Lavalette distribution to real data, coming from natural and social phenomena, and we discuss a goodness of fit test to prove that this distribution is consistent with the data. Finally, we propose a method for discerning between Lavalette and lognormal distributions and discuss the implications of our findings.

## Results

The two representations of a distribution, pdf and rank-frequency plot, can be converted from one to the other in these two ways: (i) equating cumulative distribution function (cdf) to reversed normalized rank: $r_{\left[x\right]}/N\;\approx \;1\;-\;\int_{-\infty }^{x}p\left(t\right)dt$; (ii) equating the averaged rank of a value *x*, 〈*r*_[*x*]_〉, to the *n* which maximizes the following probability: $\left(N-n+1\right)\left(\begin{array}{c} N\\ n \end{array}\right){\left(\int_{-\infty }^{x}p\left(t\right)dt\right)}^{N-n}\cdot p\left(x\right)\cdot {\left(\int_{x}^{\infty }p\left(t\right)dt\right)}^{n-1}$ [2]. Below, we will only use (i) in deriving a relationship between the pdf and the rank-frequency representations.

The Lavalette rank function:

$$x_{\left[r\right]}=C\;{\left(\frac{N+1-r}{r}\right)}^{a}$$ (2)

can be converted to

$$\frac{r_{\left[x\right]}}{N}=\frac{N+1}{N}\frac{1}{1+{\left(\frac{x}{C}\right)}^{1/a}}\approx \frac{1}{1+{\left(\frac{x}{C}\right)}^{1/a}},$$ (3)

with the right-hand-side being 1-cdf. The pdf is then the negative derivative of Eq (3).

### The Lavalette Distribution

A certain quantity *X* follows a Lavalette rank function if its rank-frequency or rank-size function is a DGBD Eq (1) with equal parameters *a* = *b* ([29]). As we saw, the pdf of *X* is proportional to the negative derivative of the inverse *r*_[*x*]_. We say that a random variable *X* has a Lavalette distribution with parameters *C* and *a* if it has the density

$$p{\left(x\right)}_{lav}=\frac{1}{aC}\frac{x^{1/a-1}}{{\left(1+{\left(\frac{x}{C}\right)}^{1/a}\right)}^{2}}.$$ (4)

**Fig 1:**
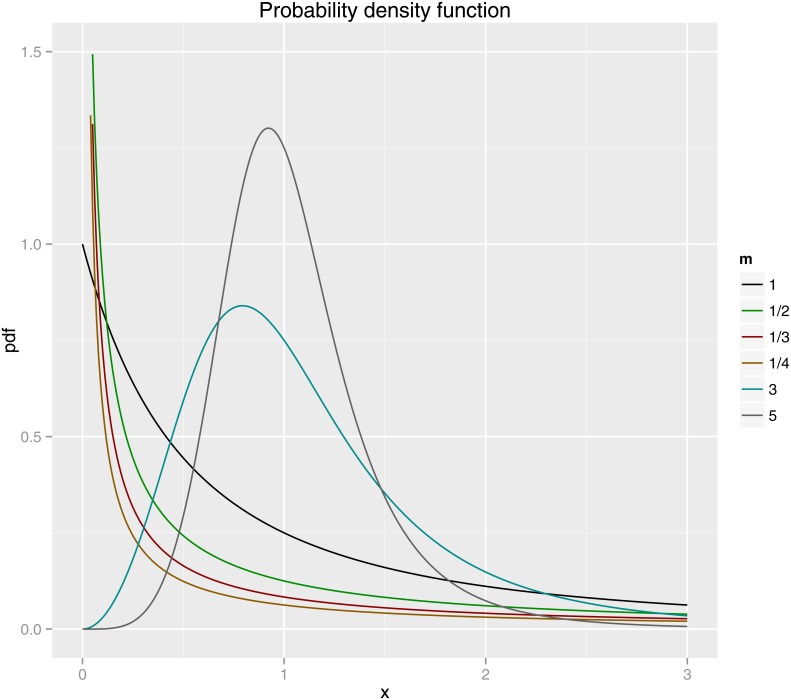
Pdf of the Lavalette distribution. Some Lavalette probability density functions (Eq (4)) with identical parameter *C* = 1 but with a = 1/5, 1/3, 1, 2, 3, and 4 (*m* = 1/*a* = 1/*b*).

With the analytic expression of Eq (4), many properties of the Lavalette distribution can be easily obtained. The *i*-th moment is:

$$\begin{array}{ll} E\left[x^{i}\right] & =\;\int_{0}^{\infty }x^{i}p\left(x\right)dx\;=\frac{C^{i}}{a}\int_{0}^{\infty }\frac{{\left(x_{1}\right)}^{1/a-1+i}}{{\left(1+{\left(x_{1}\right)}^{1/a}\right)}^{2}}dx_{1}\\ & =\;iC^{i}\int_{0}^{\infty }\frac{{\left(x_{1}\right)}^{i-1}}{1+{\left(x_{1}\right)}^{1/a}}dx_{1}\\ & =\;aiC^{i}\int_{0}^{\infty }\frac{{\left(x_{2}\right)}^{ai-1}}{1+x_{2}}dx_{2}=\frac{aiC^{i}\pi }{\text{sin}\left(ai\pi \right)} \end{array}$$ (5)

($x_{1}=x/C,x_{2}=x_{1}^{1/a}$) (if *i* < 1/*a*) (see, e.g., [34] or http://en.wikipedia.org/wiki/List_of_definite_integrals). In particular, the mean of a Lavalette random variable is

$$E\left[x\right]=\pi aC\sin{\left(\pi a\right)}^{-1},$$

which is finite if *a* < 1, while its variance is

$$Var\left[x\right]=\pi aC^{2}\left(2\sin{\left(2a\pi \right)}^{-1}-\pi a\sin{\left(a\pi \right)}^{-2}\right),$$

which exists and is finite if *a* < 1/2. However, similar to the discussion of power law distributions, whether the moments diverge to infinity or do not depends on whether a lower bound of the functional form is imposed [1]. One may re-derive the connection between ranked data and pdf by $r_{\left[x\right]}/N=1-\frac{\left(N-1\right)}{N}\int_{x_{m}}^{x}p\left(t\right)pt/\int_{x_{m}}^{x_{M}}p\left(t\right)dt$ where *x*_*m*_ and *x*_*M*_ are the minimum and maximum values among *N* samples. Fig 1 shows a plot of the Lavalette density for different parameters: they all have identical *C* = 1 but *a* = *b* = 1/3, 1/5 (unimodal) and *a* = *b* = 1, 2, 3, 4 (monotonically decaying).

### Resemblance between Lavalette and lognormal distributions

**Fig 2:**
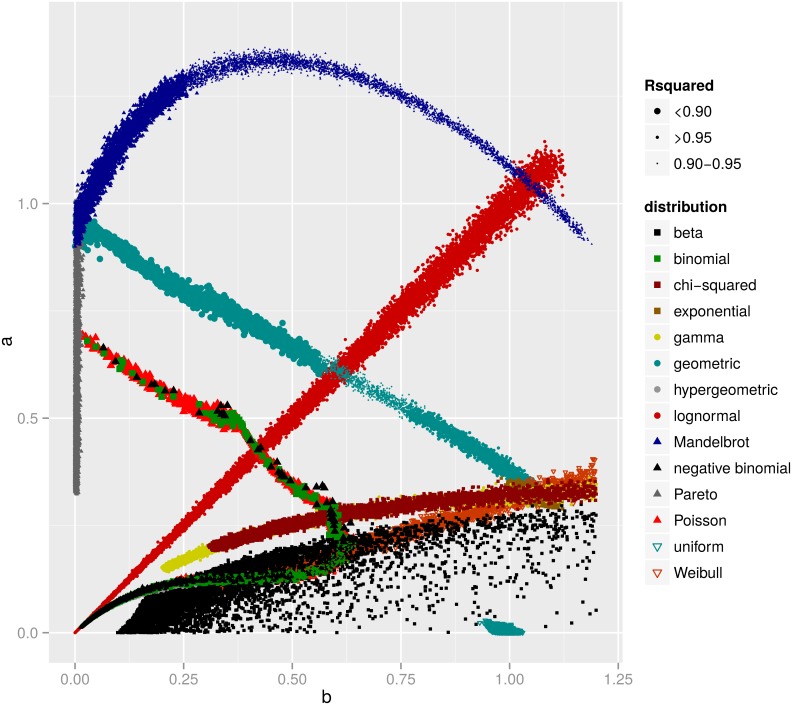
DGBD fits for various distributions. The estimated *a* and *b* parameter values in DGBD (Eq (1)) for data generated by well known distributions. Size of the dots indicate the coefficient of determination R squared. The dots around the *a* = *b* diagonal line are for data generated by the lognormal distribution.

To examine which well known pdf’s share the same property of *a* = *b* when fitted to the DGBD rank function, we generated data from 14 distributions (beta, binomial, *χ*^2^, exponential, gamma, geometric, hypergeometric, lognormal, Mandelbrot, negative binomial, Pareto, Poisson, uniform and Weibull), and fit the ranked data by the Beta rank function via linear regression of the logarithmic transformation of Eq (1). The estimated parameter values for *a* and *b* are shown in Fig 2. Interestingly, the only known pdf which exhibits *a* ≈ *b* is the lognormal distribution.

We use a novel argument from statistics to explain why the Lavalette and lognormal distributions may be difficult to distinguish within a certain interval of their domain. There are two models for probability of a binary variable *y* ∈ (0,1): (i) probit model [35]: *P* = *P*(*y* = 1) = Φ(*z*) where Φ is the cdf of standard normal distribution; (2) logit model or logistic regression [36]: *P* = 1/(1+*e*^−*z*^). The two regression models for binary variable (regressed over an independent variable *z*) usually lead to similar results [37, 38], which can be written as (after the logistic variable being re-scaled by a factor *α*):

$$\Phi \left(z\right)\;\approx \frac{1}{1+e^{-\alpha z}},\;\text{o}r,\frac{\Phi (z)}{1-\Phi (z)}\approx e^{\alpha z}.$$ (6)

The *α* can be $\sqrt{8/\pi }\approx 1.596$ to achieve the best fit near the midpoint [39], or ≈ 1.7 to best fit the whole range, or $\pi /\sqrt{3}\approx 1.81$ which is the standard deviation of the variable from the logistic distribution [38]. The standard normal variable can be converted to a lognormal distribution variable *x*: *z* = (log(*x*) − *μ*)/*σ*, and re-expressing Eq (6) in *x* becomes:

$$e^{\mu }{\left(\frac{\Phi ((\log(x)-\mu )/\sigma }{1-\Phi ((\log(x)-\mu )/\sigma )}\right)}^{\sigma /\alpha }\approx x,$$ (7)

which we recognize as the Lavalette rank function over variable *x* (1 − Φ is the normalized rank). This derivation also points out that the parameter *a* = *b* is the standard deviation of the lognormal distribution divided by *α*(= 1.6 ∼ 1.8), whereas the log-mean of the lognormal distribution is related to the scaling parameter by *C* = *e*^*μ*^. Since probit and logistic regression are not the same, we conclude that the Lavalette and the lognormal distributions cannot be identical. Indeed, the Lavalette and lognormal distributions have qualitatively different behaviors at the tails. All moments of the lognormal distribution exist, while the Lavalette has only finite moments of order *i* < 1/*a*, as we previously discussed. If there is enough data to sample the tail, they cannot be mistaken into one another.

**Fig 3:**
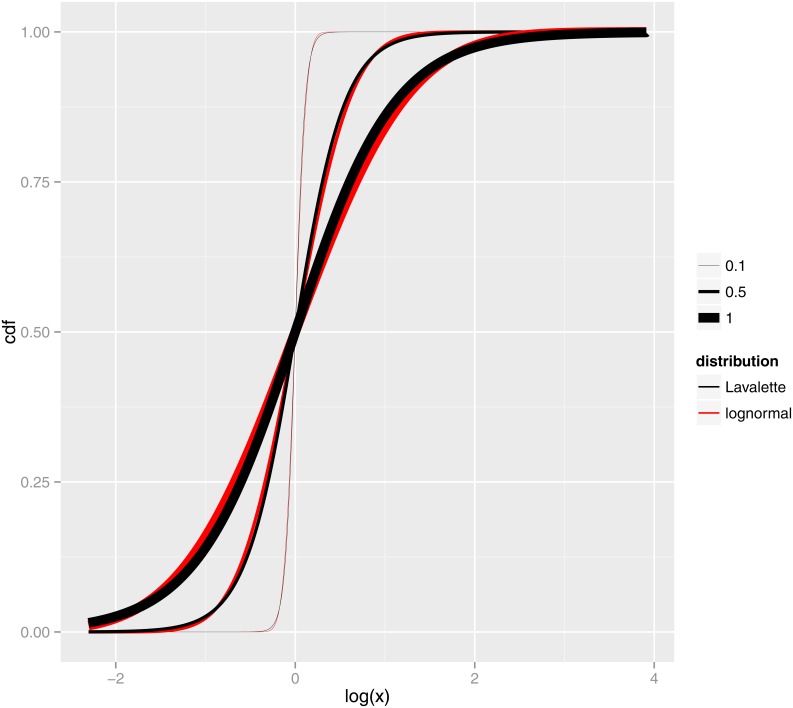
Lognormal vs Lavalette cdf. Cumulative distribution function for lognormal and Lavalette distributions, being *μ* = 0 and *σ* = 0.1, 0.5 and 1 the parameters of the lognormal. The *x* axis is in logarithmic scale. We see that over an important interval of the domain, it may be difficult to distinguish a lognormal from a Lavalette distribution.

Fig 3 illustrates directly the similarity between the Lavalette and lognormal distributions. The cdf’s of lognormal distribution and the corresponding Lavalette distribution are plotted at three different parameter values (*μ* = 0 with *σ* = 0.1, 0.5 and 1 for the lognormal, corresponding $C=e^{\mu },\;a=\sigma \sqrt{3}/\pi$ for the Lavalette). Besides the difference at the tails (which is not visible from the cdf plot because the difference along the *y*-axis is very small for extreme values), the two functions also deviate slightly from each other in the middle range. This deviation is equivalent, after a transformation, to that between the cdf of standard normal distribution and logistic function. It has been proposed that a modification of the logistic function, 1/(1 + *exp*(−1.5876*x* − 0.070566*x*^3^)), is a very good approximation of the cdf of standard normal distribution [39, 40]. The small coefficient of the high-order term is another indication that the cdf of normal and logistic function, or equivalently, lognormal and Lavalette distributions, are close.

### Occurrence and Applications

To illustrate that Lavalette distribution can be applied to real data, we examine several datasets besides the impact factor and citation data used in [29, 30]. We will give examples of population data, amino acid mutation rates and codon usage data where the Lavalette distribution is a good statistical model. The parameters were estimated through linear regression of the logarithmic transformation of Eq (1), which in our case gives very similar results to maximum likelihood estimators. The goodness of fit tests were performed using the Kolmogorov-Smirnov statistic and the *p*−values were estimated through a Monte-Carlo approach proposed in ([1]). As usual, a small *p*− value leads to reject the hypothesis that the data are well described by a Lavalette distribution.

**Fig 4:**
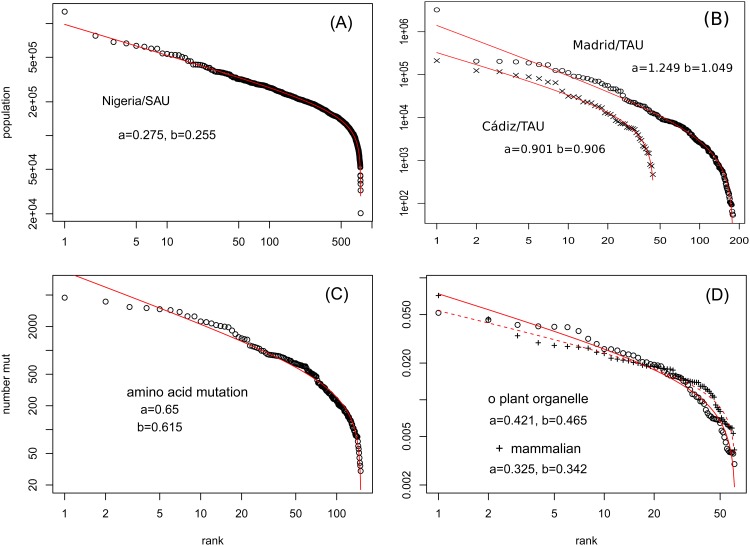
Ranked datasets fitted by Lavalette rank function. (A) Nigeria (NRG) local government area (the secondary administrative unit (SAU)) population; (B) Madrid and Cádiz municipality (the tertiary administrative unit (TAU)) population; (C) Amino acid to amino acid mutation counts in the 1000 Genomes Project; (D) Averaged codon usage (excluding the three stop codons) of plant organelles and mammals.

The first set of examples is about administrative units of population. Most countries in the world are internally divided into administrative units, which may be called states, provinces, etc. [41]. We call these primary administrative units (PAU), which may be in turn subdivided into smaller or second level administrative units (counties, municipalities, etc.) We call these secondary administrative units (SAU). In the same way, there may be third level units (TAU) and so forth. We give three examples of occurrence of the Lavalette distribution: the Nigeria (NRG) population of local government areas (SAU) and the municipality population (TAU), below province and autonomous community, within the Spanish provinces of Madrid and Cádiz. We chose these examples after analysing population data from many countries in the world and picking those that are best fitted by the Lavalette function. We emphasize that we do not claim the Lavalette distribution to be ubiquitous in any way; our purpose is to show that there are some datasets where it can be a good statistical model.

(Fig 4A and 4B) shows the rank frequency distribution of the NRG/SAU, Madrid/TAU and Cádiz/TAU population in log-log scale. The fitted parameter values (*a*, *b*) by Eq (1) are (0.275, 0.255) for NRG/SAU, (1.249, 1.049) for Madrid/TAU, (0.901, 0.906) for Cádiz/TAU, all with *a* ≈ *b*. Clearly these do not follow a power law distribution. Although city population is one of the well known examples of Zipf’s law [42, 43], there is a difference between cities and administrative units. The origin of Zipf’s law in population and economic phenomena might be explained by a proportionate-growth random process [4]. For the particular case of well separated cities, as well as firm sizes, birth and death processes explain the origin and robustness of Zipf’s law [44]. However, when regions are artificially partitioned, such as the case of administrative units, the argument for power-law may fail. Indeed, the bad fitting performance of Zipf’s law on data in some counties [45, 46] might be caused by the artificial boundary in defining a city [47]. This leaves room for alternative functional form such as DGBD. [17].

The second example is the amino acid mutation rates [48] based on the amino acid changing (missense) variants in the 1000 Genomes Project [49]. A missense mutation is a point mutation which results in the codification of a different amino acid. Because the variants are observed in normal human population with a short evolutionary history, it can be considered as an instantaneous mutation rate. The substitution rate between different species, such as the point accepted mutation (PAM) [50], cover a much longer evolutionary history with stronger selection constraints. Out of 380 (= 20 × 19) possible mutations between 20 amino acids, only *N* = 150 are allowed from the single base mutation in the DNA sequence, due to the nature of the genetic code. Fig 4(C) shows the ranked amino acid to amino acid frequencies derived from the missense variants in DNA sequence of the 1000 Genomes Project. Fig 4(C) shows a fitting by the Beta rank function Eq (1) with *a* ≈ 0.650 and *b* ≈ 0.615, which is again a good Lavalette function.

The third example is the codon usage of *N* = 61 non-stop codons, with data from the Codon Usage Database [51]. Codon usage refers to the frequency of occurrence of 181 each type of codon within a DNA sequence. We picked the two examples best demonstrating Lavalette function: genes in plant organelles (9221 species) and in (non-primate, non-rodent) mammalian nucleus (433 species). The codon frequencies are averaged over all species in plant organelle and mammalian separately. The three stop codons are discarded. The (*a*, *b*) are (0.422, 0.465) for plant organelles, and (0.325,0.342) for mammalian (Fig 4(D)).

With the previous examples we have illustrated the occurrence of the Lavalette distribution. Next we propose a statistical criterion to discern if this distribution is consistent with the data.

### Goodness of Fit Tests

The first clue that a certain dataset may be well described by the Lavalette distribution is to fit the data to DGBD function Eq (1), estimate the parameters and check if *a* ≈ *b*. If this is the case, the data set is a candidate for the Lavalette distribution. This is a first criterion and it serves to rule out many datasets; however, it is by no means strong statistical evidence to claim the the Lavalette is a good model for the data.

To test more rigorously whether a Lavalette function fits the observed data well, we use a re-sampling approach as discussed in [1] which can also be called a *bootstrap* [52]. We first fit the data by the Lavalette function (Eq (2)). The difference between the observed and fitted value is measured by the Kolmogorov-Smirnov (KS) distance. Using the fitted Lavalette rank function, artificial data (replicates) are generated multiple times: each time a new Lavalette rank function is fitted and KS distance calculated. The proportion of replicates with larger KS distances than the observed one is the empirical *p*-value.

A large empirical *p*-value indicates that there is not enough evidence to reject the Lavalette function. Empirical *p*-values from 1000 replicates are 0.49 for NRG/SAU, 0.91 for Madrid/TAU, 0.88 for Cádiz/TAU, 0.06 for mutation rate, and 0.4 for codon usage in both plant organelle and mammals. These values depend on many specific choices used, e.g. how to handle replicates which have the same KS distance as the observed one, using KS distance instead of some other measure of difference between two curves, the number of replicates, etc. The empirical *p*-value we have indicate that Eq (2) is a good fitting function for these data.

There have been debates in the literature whether Zipf’s law results from the central limit theorem [53–55]. Given a dataset, the best answer to that debate is to pick the better fitting model between power law and lognormal distribution [56]. The approximate equivalence between Lavalette distribution and lognormal distribution provides us with a simple method in deciding if a set of data follows Zipf’s law or lognormal distribution. For the fitting of ranked data by the Beta rank function Eq (1), if *b* ≈ 0, the Zipf’s law is better; if *a* ≈ *b* > 0, lognormal is better; and if *a* ≠ *b* ≫ 0, neither are good fitting functions.

For our examples to illustrate the Lavalette distribution in real data, it is obvious that lognormal distribution is a better fitting function than the Zipf’s law. We can further quantify the fitting performance by model selection techniques such as Akaike information criterion (AIC) [57–59], with the better model exhibiting lower AIC value. The AIC_*lav*_-AIC_*zipf*_ = *N* log(*SSE*_*lav*_/*SSE*_*zipf*_) [18], where SSE is the sum squared error, is -3284.6,-410.3, -108.1 for the NRG/SAU, Madrid/TAU, Cádiz/TAU data, -353.7 for the amino acid mutate data, and -101.7, -114.7 for plant organelle, mammalian codon frequencies, all representing an overwhelming support to the Lavalette function or lognormal distribution over the Zipf’s law.

## Discussion and Conclusions

We have presented a novel probability distribution function and showed that it is a good alternative for data that does not follow a perfect Zipf’s law. We have seen that this distribution yields a very good approximation to the lognormal distribution. Although it is perhaps less important because of the approximate equivalence between the Lavalette and the lognormal distributions, one may still sometimes want to determine whether a data is better fitted by the Lavalette or lognormal distribution. We propose the following procedure for this test: (i) log-transform, then standardize (zero mean, unit standard deviation) the raw data, to *x*′; (ii) compare the empirical cdf of *x*′ to both standard normal and logistic distribution cdf with a scaling parameter $\alpha =\pi /\sqrt{3}$ (*cdf* = 1/(1 + *e*^−*αx*′^)); (iii) if the standard normal function is closer to the data, lognormal distribution fits the original data better, otherwise, Lavalette function is better. Using this procedure and KS distance as the measure of difference, NRG/SAU is the only data which Lavalette is better than lognormal distribution. If sum of absolute error is used to measure the difference, codon usage of mammals is another data which prefers Lavalette over lognormal.

The Lavalette distribution may have useful applications in spatial ecosystems. Formation of spatial patterns is a topic of growing relevance in theoretical ecology and populations dynamics [60]. For example, deviations from power laws in vegetation patchiness occur in ecosystems that are close to desertification [61]; in predator-pray systems, spatial patterns with a large isolation degree are related with the collapse of the ecosystem [62]. Because the Lavalette distribution adequately characterizes data that deviate from power laws, we believe it may find an important field of applications in spatial ecology by describing and quantifying power-law-like behaviors and representing systems near a critical transition. This is a matter of future study.

In conclusion, by connecting Lavalette function to lognormal distribution, we achieve a better understanding of the DGBD function and the limitations of the Zipf’s law.

## Materials and Methods

Population data for administrative units and sub-units in a large sample of countries is available in the database Statoids http://www.statoids.com (accessed April 2016). Population of local government areas (SAU) in Nigeria were taken from this database. Spain’s population at PAU, SAU and TAU levels are available from the National Statistics Institute (INE), http://www.ine.es/en/pob_xls/pobmun12_en.xls. We chose these examples after analysing population data from many countries in the world and picking those that are best fitted by the Lavalette function.

Amino acid to amino acid mutation rates were calculated from missense variants, taken from DNA sequence of the 1000 genomes project, available at http://www.1000genomes.org/. In particular, we used mutation data from http://journals.plos.org/ploscompbiol/article?id=10.1371/journal.pcbi.1003382 Fig (1). From this data, we counted the relative frequency of occurrence for each mutation.

We calculated codon usage of 61 non-stop codons for genes in plant organelles and non-primate and non-rodent mammalian nucleus. Data were downloaded from Codon Usage Database http://www.kazusa.or.jp/codon/.

All the data used in our analysis is available on https://figshare.com/articles/Data_rar/3363961.

Each data set was fitted to a Lavalette distribution and the parameters were estimated by a linear regression model of the logarithmic transformation of Eq (1) with *a* = *b*. We tested the goodness of the fit of the Lavalette model by using the re-sampling approach to the Kolmogorov- Smirnov test proposed in [1]. Finally, we compared the performance of the Lavalette and the power law models by using the Akaike Information Criterion. All these methods are more carefully described in section *Occurrence and Applications*.

## Supporting Information

S1 File Examples of occurrence of the Lavalette distribution. The file S1_File.rar contains the data we used to illustrate the occurrence of the Lavalette distribution. The files nigeria-tau.txt and spain-tau.txt consist of the population data sets of Spain and Nigeria analyzed in this work. Missense variants utilized to calculate amino-acid to amino-acid rates are in the file 1000G-matrix.txt. The files mammalian-no-organelle.txt and plant-organelle.txt contain the codon usage information in mammalian and plant organelle nucleus respectively.
(RAR)
